# Retinal transcriptome of neonatal mice after optic nerve injury

**DOI:** 10.1371/journal.pone.0286344

**Published:** 2023-05-30

**Authors:** Shi-Qi Yao, Meng Wang, Jia-Jian Liang, Tsz Kin Ng, Ling-Ping Cen

**Affiliations:** 1 Joint Shantou International Eye Center of Shantou University and The Chinese University of Hong Kong, Shantou, Guangdong, China; 2 Shantou University Medical College, Shantou, Guangdong, China; 3 Department of Ophthalmology and Visual Sciences, The Chinese University of Hong Kong, Hong Kong, China; University of Dayton, UNITED STATES

## Abstract

**Background:**

The axonal growth capacity of retinal ganglion cells decreases dramatically within the first day of birth, and the axonal regeneration after injury in mature mammals is very limited. Here, this study aimed to delineate the transcriptomic changes associated with altered axonal growth capacity and to identify the key genes associated with axonal regeneration by the RNA sequencing (RNA-Seq) analysis.

**Methods:**

The whole retinas from the mice of embryonic day (E) 20, postnatal day (P) 1 and P3 were collected at 6 hours after optic nerve crush (ONC). Differentially expressed genes (DEGs) for ONC or ages were identified by the RNA-Seq analysis. *K*-means analysis was conducted for the clustering of DEGs based on expression patterns. Enrichment of functions and signaling pathways analysis were performed based on Gene Ontology (GO), Kyoto Encyclopedia of Genes and Genomes (KEGG) database, and Gene Set Enrichment analysis (GSEA). Quantitative real time polymerase chain reaction (qRT-PCR) was used to validate the DEGs selected from the RNA-Seq analysis.

**Results:**

In total, 5,408 DEGs were identified for ages, and 2,639 DEGs in neonatal mouse retina after ONC. *K*-means analysis revealed 7 clusters in age-DEGs and 11 clusters in ONC-DEGs. The GO, KEGG and GSEA pathway analyses identified significantly enrichment of DEGs in the visual perception and phototransduction for the age effect, and the break repair, neuron projection guidance, and immune system pathway for the ONC. PPI analysis identified hub genes in the axon-related gene cluster. The expressions of *Mlc1*, *Zfp296*, *Atoh7*, *Ecel1*, *Creb5*, *Fosb*, and *Lcn2*, thought to be involved in RGC death and axonal growth were validated by qRT-PCR.

**Conclusions:**

This study, for the first time, delineated the gene expression changes following ON injury in embryonic and neonatal mice, providing a new resource of age- and injury-driven data on axonal growth capacity.

## Introduction

Axons of retinal ganglion cells (RGCs) form the optic nerve (ON), conveying the visual information from the retina to the visual cortex in the brain [1]. Optic nerve injury causes the death of ~80% of RGCs within 2 weeks in mice [2]. As the central nervous system (CNS) neurons in mature mammals, axonal regeneration in RGCs is very limited once they are injured. Traumatic, ischemic or degenerative conditions of ON injury, such as glaucoma, result in irreversible loss of vision. Failure in axonal regeneration after injury is mainly due to the poor intrinsic regenerative capacity of neurons and multiple inhibitory factors from the surrounding glial environment [1].

During the initial formation of visual projections, most of the RGCs start to differentiate between embryonic day 13 (E13) and E18, extending their axons towards their targets between E14 and postnatal day (P2) [3], and are able to regenerate the injured axons over a short distance *in vivo*, but the ability of axonal growth and regeneration rapidly lost in the early stage after birth [4]. This change in intrinsic growth capacity is accompanied by substantial changes in gene expression programs [4]. Comprehensive analysis of the transcriptome helps to understand the molecular events in the retina during development [5].

Research over the past 20 years has shown that, in some cases, mature RGCs can be transformed into an actively regenerating state, allowing these neurons to survive and regenerate the axons [6]. RNA sequencing (RNA-Seq) is able to identify the genes involved in the regeneration of axonal injury after ON crush (ONC); however, most of the studies are typically based on the adult mice [2, 7], and some key genes may undergo loss of regenerative capacity early in the development. Herein this study aimed to determine the retinal transcriptome at 6 hours after ON crush (ONC) in embryonic and neonatal mice by RNA-Seq in order to identify the changes in comprehensive gene expression after axonal injury in the early stage of development.

## Materials and methods

### Ethics statement

All animal experiments were conducted following the Statement on the Use of Animals in Ophthalmic and Vision research from Association for Research in Vision and Ophthalmology, and approved by the Animal Experimentation Ethics Committee of the Joint Shantou International Eye Center of Shantou University and the Chinese University of Hong Kong (approval no.: EC20150313(1)-P06) on March 13, 2015.

### Animals

All the pregnant C57BL/6 mice (age: 8–12 weeks; average weight: 20–27 g) were purchased from Beijing Charles River Laboratory Animal Technology Co. Ltd., China. Each pregnant mouse was housed in a separate cage and kept in standard condition with a 12-hr dark/light cycle at 21–23°C, and fed with food and water *ad libitum* until the E20 or naturally delivery.

Three age groups of the mice were chosen for the experiments: E20, P1, and P3. Mice of the same age group were born in the same litter. 3–4 mice were used for each group, and each retina sample was considered as a biological replicate for the gene expression study. After the ONC, the neonatal mice were euthanized by cervical dislocation.

### Optic nerve crush

The pregnant mice were anesthetized by a mixture of xylazine (20 mg/ml) and ketamine (100 mg/ml) based on their body weight (1.0 ml/kg), and sacrificed by cervical dislocation. Embryonic and newborn mice were placed on ice until they were anesthetized, and the operations were performed on ice. The ON was crushed at 0.5 mm behind the eyeball for 5 sec using an angled jeweler’s forceps (Dumont #5; Roboz) [8]. Damage to the ophthalmic artery beneath the ON was avoided. Effective ON injury was confirmed by the presence of a clear gap across the entire ON at the crush site. The whole process was carried out under anesthetized. After the surgery, the mice were placed on the 37°C thermostat plate (Roland Consult, Brandenburg, Germany) for 6 hours to ensure their survival.

### RNA preparation

At 6 hours after the ONC surgery, the mice were placed on ice until they were anesthetized, and the retinas were immediately dissected out from the enucleated eyeballs in phosphate buffered saline (PBS), then the mice were sacrificed by cervical dislocation. The retinas were homogenized with the TRIzol Reagent (Thermo Fisher Scientific), and total RNA was extracted from the homogenized mixture according to the instructions. Total amounts and integrity of RNA was assessed with the RNA Nano 6000 Assay Kit using the Bioanalyzer 2100 system (Agilent Technologies).

### Library preparation and sequencing

RNA sequencing experiments were performed by Novogene, Inc. (Beijing, China). Briefly, total RNA of the samples from three biological replicates first underwent mRNA purification with poly-T oligo-attached magnetic beads. Sequencing libraries were generated using NEBNext^®^ UltraTM RNA Library Prep Kit for Illumina^®^ (NEB) following the manufacturer’s recommendations, and the index codes were added to attribute the sequences to each sample. The library fragments were purified with the AMPure XP system (Beckman Coulter) for the cDNA fragments of preferentially 370–420 bp in length. The library quality was assessed using the Agilent Bioanalyzer 2100 system. After the library was qualified, different libraries were pooled according to the effective concentration and the target amount of data off the machine, and sequenced by the Illumina NovaSeq 6000. The end reading of 150 bp pairing was generated.

### Gene expression analysis

The RNA-Seq reads were aligned to the reference genome using Hisat2 v2.0.5. Feature Counts v1.5.0-p3 was used to count the reads numbers mapped to each gene. The Fragments Per Kilobase of transcript sequence per Millions base pairs sequenced (FPKM) of each gene was calculated based on the length of the gene and reads count mapped to this gene, which normalizes the gene expression by considering the effect of sequencing depth and gene transcript length at the same time. RNA-seq datasets of mouse retina generated in this work are available at GEO (GSE232373). Differential expression analysis was performed using the DESeq2 R package (1.20.0). The *p*-value were adjusted using the Benjamini and Hochberg’s approach for controlling the false discovery rate (less than 0.1). The genes with adjusted *p* < 0.1 and fold change (FC) > 1.5 (detected by DESeq2) were considered as the differentially expressed genes (DEGs).

### *K*‐means and enrichment analyses

*K*‐means clustering of unions of the DEGs was performed using the ggplot2 and pheatmap R package (3.0.3) [9]. Based on the fuzzy *K*‐means algorithm, 6‐20 *K*‐means clusters were divided and an optimal one was selected for DEGs. Enrichment analysis was conducted on every cluster of genes. Gene Ontology (GO) and Kyoto Encyclopedia of Genes and Genomes (KEGG) analyses of DEGs were conducted using the Cluster Profiler R package (3.8.1). The GO functional enrichment analysis including the biological process (BP), cellular component (CC), and molecular function (MF). *P* < 0.05 were considered as significantly enriched by the DEGs.

### PPI and GSEA analyses of differentially expressed genes

The DEGs were integrated with mouse protein-protein interaction (PPI) networks using STRING. For the species that exist in the database, we build a network by extracting the list of target genes from the database. Otherwise, the Diamond software (0.9.14) was used to compare the target gene sequence with the selected reference protein sequence, and then the network was established according to the known interaction of the selected reference species.

Gene Set Enrichment analysis (GSEA) is a statistical method that use a predefined gene set to sort genes according to the degree of differential expression in a sample, and then test whether the predefined gene set is enriched at the top or bottom of this sorted table [10]. GSEA and ClusterProfiler [4.4.4] were used to analyze the path and define each functional cluster, and false detection rate (FDR) < 0.25 and *P* < 0.05 was considered as significant enrichment.

### Quantitative RT-PCR

Total RNA was isolated and treated with RNase-free Dnase I (Qiagen) according to the manufacturer’s protocol. Total RNA (1μg) was reverse-transcribed into cDNA using SuperScript III reverse transcriptase (Invitrogen). Gene expressions were determined by SYBR green PCR (Roche) in the LightCycle 480 II real-time PCR apparatus (Roche) with specific primers (S1 Table) according to the manufacturer’s protocol. Automated threshold cycles (Ct) were caculated and normalized to that of the *Actb* as reference gene. The data was presented as the 2^-ΔΔCt^ values.

### Statistical analysis

The means of the data from mice ± SD were calculated. One-way ANOVA with post hoc Bonferroni test (for multiple testing correction) was used to compare the means among different treatment groups. All statistical analyses were performed using commercially available software (IBM SPSS Statistics 21; SPSS Inc). Significance was defined as *P* < 0.05.

## Results

### Expression analysis

**Fig 1:**
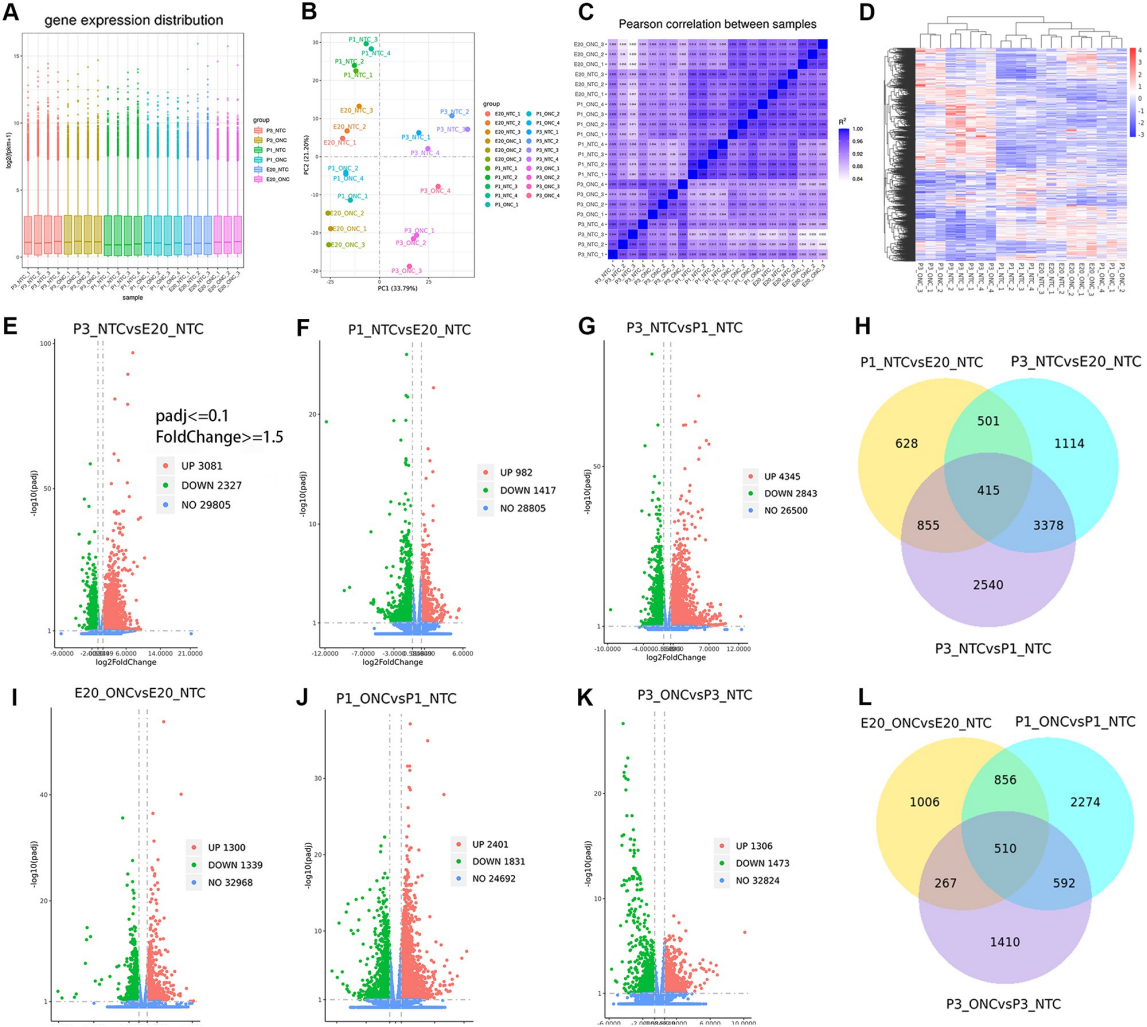
Quantitative analysis of retinal gene expression. **(A)** Boxplot shows the overall range and distribution of FPKM value of gene expression of all the samples. **(B)** PCA analysis shows the differentiation among all the samples. **(C)** Pearson correlation between all the samples. The more correlation coefficient closer to 1, the more similar the expression pattern between the samples. **(D)** Cluster analysis of genes among samples. The color of the heat map indicated the relative gene expression. **Differentially expressed genes analysis. (E-G, I-K)** The volcano map shows the differentially expressed genes in different comparison groups. **(H)** The Venn diagram showing the common regulated DEGs during the develop of E20-P1-P3. **(L)** The Venn diagram showing the overlapping regulated DEGs after ONC.

Three samples were used in each group, and each sample contained only one retina. Sample total reads ranged from 41.4 to 54.7 million, and mapping rate ranged from 86.2% to 92.9%. The boxplot result showed that the overall distribution of the FPKM values were similar in each sample, suggesting that the RNA-seq data were reproducible (Fig 1A). The two-dimensional Principal component analysis (PCA) showed that the ONC samples were separated from the samples without ONC at each time points (E20/P1/P3), and different age groups also separated from each other (Fig 1B). The PCA of different age groups revealed the transcriptome differences caused by the developmental differences. Largest difference was found in the expression patterns between the P3 and E20 (Fig 1C and 1D), suggesting that the most dramatic gene changes happened.

### Differentially expressed genes analysis

The triplicated samples from the P3_NTC and E20_NTC groups were assayed for DEGs to identify the candidate genes regulated by ages that 3,081 upregulated and 2,327 downregulated genes were identified (Fig 1E), indicating the great alterations in gene expression during retina development from E20 to P3. In order to narrow down the most promising candidate DEGs, we compared the DEGs identified in E20 to P3 with P1. The results suggested that 2,399 genes (982 upregulated and 1417 downregulated) were found in P1 as compared to E20 (Fig 1F), while 7,188 genes (4345 upregulated and 2843 downregulated) were found in P3 as compared to P1 (Fig 1G). Only 415 genes were commonly regulated (Fig 1H) from the transition of E20-P1-P3(ages-DEGs). The 10 most upregulated and downregulated genes are listed in Table 1.

**Table 1:** Top 10 up- and downregulated genes.

Gene_Name	Description	TF_family	Log_2_ FC	*P*-adj
**Up-regulated (P3_NTC vs E20_NTC)**				
*Cnga1*	cyclic nucleotide gated channel alpha 1	-	10.31	8.03E-27
*Rs1*	retinoschisis (X-linked, juvenile) 1 (human)	-	9.35	1.00E-40
*Pax7*	paired box 7	PAX	8.87	3.68E-40
*Impg1*	interphotoreceptor matrix proteoglycan 1	-	8.61	7.06E-24
*Cryaa*	crystallin, alpha A	-	8.48	2.85E-05
*Slco1a4*	solute carrier organic anion transporter family, member 1a4	-	8.38	3.17E-12
*Rho*	rhodopsin	-	7.56	1.57E-97
*Ttr*	transthyretin	-	7.48	2.51E-21
*Gngt1*	guanine nucleotide binding protein (G protein), gamma transducing activity polypeptide 1	-	6.92	1.09E-22
*Guca1a*	guanylate cyclase activator 1a (retina)	-	6.74	5.73E-48
**Down-regulated (P3_NTC vs E20_NTC)**				
*Sox14*	SRY (sex determining region Y)-box 14	HMG	-5.77	4.91E-06
*Fgf3*	fibroblast growth factor 3		-5.00	5.12E-35
*Aldh1a3*	aldehyde dehydrogenase family 1, subfamily A3		-4.64	3.07E-10
*Fgf15*	fibroblast growth factor 15	-	-4.31	8.92E-23
*Dio3os*	deiodinase, iodothyronine type III, opposite strand	-	-4.07	6.35E-27
*Gm39119*	predicted gene, 39119	-	-4.06	7.06E-19
*Ecel1*	endothelin converting enzyme-like 1	-	-3.37	8.63E-18
*Atoh7*	atonal bHLH transcription factor 7	bHLH	-3.13	2.06E-17
*Irx2*	Iroquois homeobox 2	Homeobox	-2.95	1.14E-13
*Aspg*	asparaginase		-2.88	8.20E-08

To evaluate the extract candidate genes associated with axonal regeneration, we compared the ONC and NTC groups in E20 and P3. 1,300 upregulated and 1,339 downregulated genes were identified in the comparison between E20_ONC and E20_NTC, and 1,306 upregulated and 1473 downregulated genes were identified in the comparison between P3_ONC and P3_NTC (Fig 1I and 1K). Because the axons cannot generate at P3 [4], we removed the part of the E20 differential gene that overlapped with P3, resulting in 1,862 regulated genes as ONC-DEGs. In the same way, we removed the part of the P1 DEGs that overlapped with P3, resulting in 3,130 DEGs (Fig 1L). Among all of these DEGs, we selected 13 genes candidates based on their description and expression layer in the retina. The role of genes other than *FosB*, *Pax2*, and *Egr1* in axonal injury has not been described in detail. The 13 regulated genes are listed in Table 2.

**Table 2:** Differential expressed genes after ONC.

Gene_Name	Description	TF_family	Log_2_ FC	*P*-adj
**E20_ONCvsE20_NTC**				
*Gstp2*	glutathione S-transferase, pi 2	-	5.27	0.003
*Lcn2*	lipocalin 2	-	4.41	0.003
*Fosb*	FBJ osteosarcoma oncogene B	TF_bZIP	2.56	0.014
*Mlc1*	megalencephalic leukoencephalopathy with subcortical cysts 1 homolog (human)	-	-3.58	0.000
*Zfp97*	zinc finger protein 97	zf-C2H2	-2.02	0.000
*Pax2*	paired box 2	PAX	-1.97	0.000
*Hnf4g*	hepatocyte nuclear factor 4, gamma	RXR-like	-1.86	0.008
**P1_ONCvsP1_NTC**				
*Cpne1*	copine I	-	3.02	0.012
*Zfp296*	zinc finger protein 296	zf-C2H2	2.63	0.010
*Egr1*	early growth response 1	zf-C2H2	2.15	5.98E-18
*Zfp692*	zinc finger protein 692	zf-C2H2	1.27	1.71E-17
*Sox1*	SRY (sex determining region Y)-box 1	HMG	-2.56	0.020
*Sall3*	spalt like transcription factor 3	zf-C2H2	-2.02	8.87E-11

### Transcription factors analysis

Since transcription factors (TFs) play important roles in both axon development and regeneration, to narrow down the candidate DEGs, we analyzed the TFs upregulated and downregulated with ages or after ONC, and resulted in 16 upregulated TFs and 16 downregulated TFs for ages, and 29 upregulated TFs and 140 downregulated TFs for ONC. The top 10 regulated genes are listed in (Table 3). Among the 201 differentially expressed TFs, most of them are involved in neuron differentiation, such as *Irx2* [11], and several signaling pathways, such as *Creb5* and *Nfatc4* in PI3K-Akt signaling pathway [12], *Pax2* in Wnt signaling pathway [13], and *Fos* in MAPK pathway [14]. In addition, *Nfkb2* and *Pax7* are involved in neural crest differentiation [15].

**Table 3:** Top 10 up- and downregulated TFs.

Gene_Name	Description	TF_family	Log_2_ FC	*P*-adj
**Regulated with ages (P3_NTC vs E20_NTC)**				
*Pax7*	paired box 7	PAX	8.87	3.68E-40
*Nrl*	neural retina leucine zipper gene	TF_bZIP	4.20	1.04E-30
*Bhlhe41*	basic helix-loop-helix family, member e41	bHLH	2.90	1.99E-16
*Creb5*	cAMP responsive element binding protein 5	TF_bZIP	2.72	9.13E-24
*Fos*	FBJ osteosarcoma oncogene	TF_bZIP	2.66	3.98E-06
*Sox14*	SRY (sex determining region Y)-box 14	HMG	-5.77	4.91E-06
*Atoh7*	atonal bHLH transcription factor 7	bHLH	-3.13	2.06E-17
*Irx2*	Iroquois homeobox 2	Homeobox	-2.95	1.14E-13
*Foxn4*	forkhead box N4	Fork	-2.76	5.74E-27
*Nfatc4*	nuclear factor of activated T cells, cytoplasmic, calcineurin dependent 4	RHD	-2.31	1.43E-16
**Regulated after ONC (E20 ONC vs NTC)**				
*Fosb*	FBJ osteosarcoma oncogene B	TF_bZIP	2.56	0.014
*Gm4981*	predicted gene 4981	Homeobox	2.47	0.028
*AW822073*	expressed sequence AW822073	Homeobox	2.18	0.003
*Nfkb2*	nuclear factor of kappa light polypeptide gene enhancer in B cells 2, p49/p100	RHD	1.49	6.30E-05
*Etv4*	ets variant 4	ETS	1.29	3.75E-10
*Kdm5d*	lysine (K)-specific demethylase 5D	ARID	-11.53	0.021
*Irx1*	Iroquois homeobox 1	Homeobox	-2.29	8.28E-10
*Spdef*	SAM pointed domain containing ets transcription factor	ETS	-2.26	0.000
*Zfp97*	zinc finger protein 97	zf-C2H2	-2.02	0.001
*Zfp960*	zinc finger protein 960	zf-C2H2	-2.00	0.001

### *K*‐means analysis of differentially expressed genes

**Fig 2:**
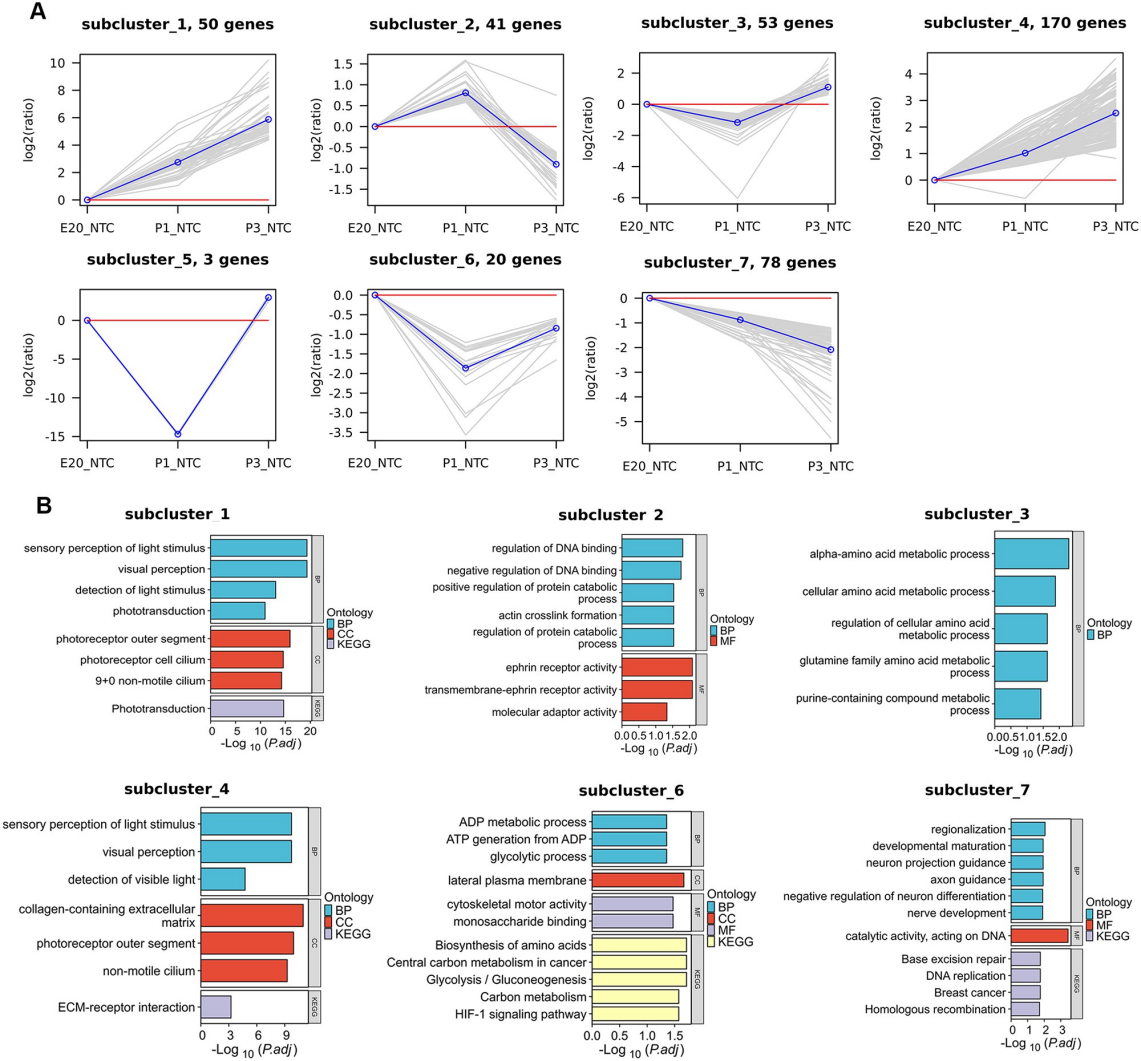
*K*-means and enrichment analyses (GO, KEGG) of age-DEGs. **(A)** *K*‐means clustering of the differential expression gene with age (*K* = 7). Each box is a DEG cluster. Each line indicates one differential expression gene. The blue line is the average trend of all DEGs in the cluster. **(B)** Gene ontology biological process term enrichment of the seven *K*‐means clusters. The top results are plotted.

**Fig 3:**
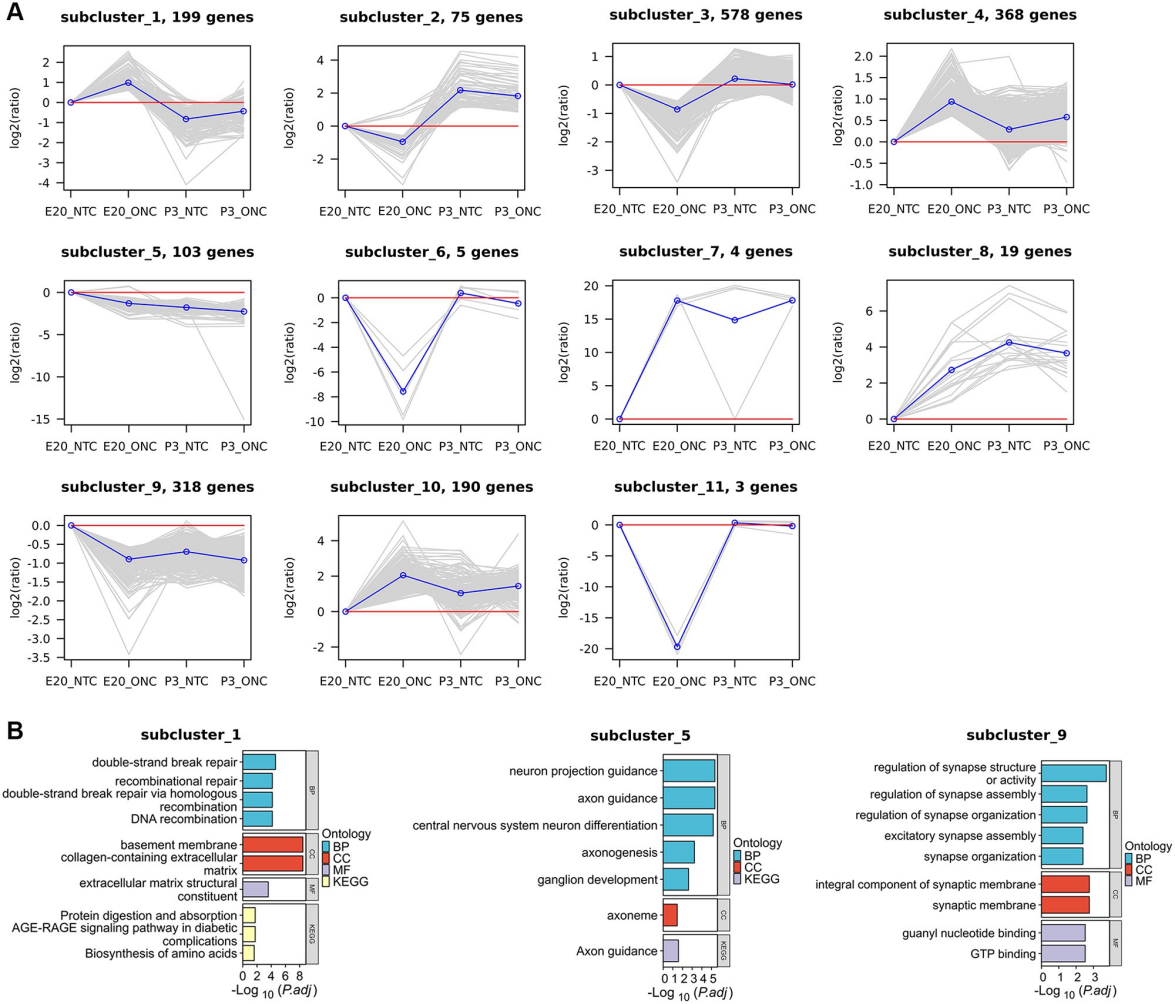
*K*-means and enrichment analyses (GO, KEGG) of ONC-DEGs. **(A)** *K*‐means clustering of the differential expression gene with age (*K* = 11). Each box is a DEG cluster. Each line indicates one differential expression gene. The blue line is the average trend of all DEGs in the cluster. **(B)** Gene ontology biological process term enrichment of the seven *K*‐means clusters. The top results are plotted.

To investigate the expression pattern of DEGs, we performed *K*‐means clustering analysis on the commonly identified 415 DEGs associated with age and 1,862 DEGs with ONC based on their group and changes. Age-DEGs were classified into 7 clusters, while 11 clusters were identified for ONC-DEGs (Figs 2A and 3A). These age-DEGs clusters were further assigned to four main groups of different dynamic patterns (Fig 2A). The first pattern group, comprising clusters 1 and 4, included 50 and 170 DEGs respectively, representing DEGs whose expression level gradually increased during development. The second pattern group, comprising of clusters 3 and 6, included 53 and 20 DEGs respectively, representing the expression level decreased in P1 then increased in P3. The cluster 2 and 7 are independent expression patterns. The pattern of cluster 2 was opposite to the second group, which increased in P1 and then decreased in P3. The expression level of cluster 7 was opposite to first group, remained decrease during development.

These ONC-DEGs clusters were assigned to two main groups of different dynamic patterns (Fig 3A). The first pattern group represents the increased expression level after ONC in E20, comprising cluster 1, 4, 7, 8 and 10. Among these clusters, only the expression level of cluster 8 was decreased after P3-ONC, while the rest were increased. The second pattern group, comprising clusters 2, 3, 5, 6, 9 and 11, and the expression level were decreased in E20-ONC. The expression levels of cluster 2 and 3 was decreased after P3-ONC, and the rest were increased.

### GO and KEGG pathway analysis

To further understanding the potential functional role of DEGs, GO and KEGG analyses were performed in each cluster. In the age-DEGs clusters, GO enrichment analysis showed that the visual perception and light stimulus was enriched in cluster 1 and 4, while “Phototransduction” was significantly enriched in the KEGG pathway analysis (Fig 2B). Metabolic-related processes were enriched in cluster 3 and 6 (Fig 2B). Moreover, the DEGs in cluster 7 were enriched in the term related to axon guidance and nerve development (Fig 2B).

We further analyzed the ONC-DEGs on the potential regeneration processes. The GO enrichment analysis showed that the DEGs in cluster 1 were mainly involved in the term related to repair (Fig 3B). Neuron projection guidance and axonogenesis were enriched in cluster 5. The KEGG pathway analysis also indicated that these DEGs were mainly enriched in the “Axon guidance” pathways (Fig 3B). In addition, synapse-related terms were mainly involved in cluster 9 (Fig 3B).

### PPI network and GSEA analysis

**Fig 4:**
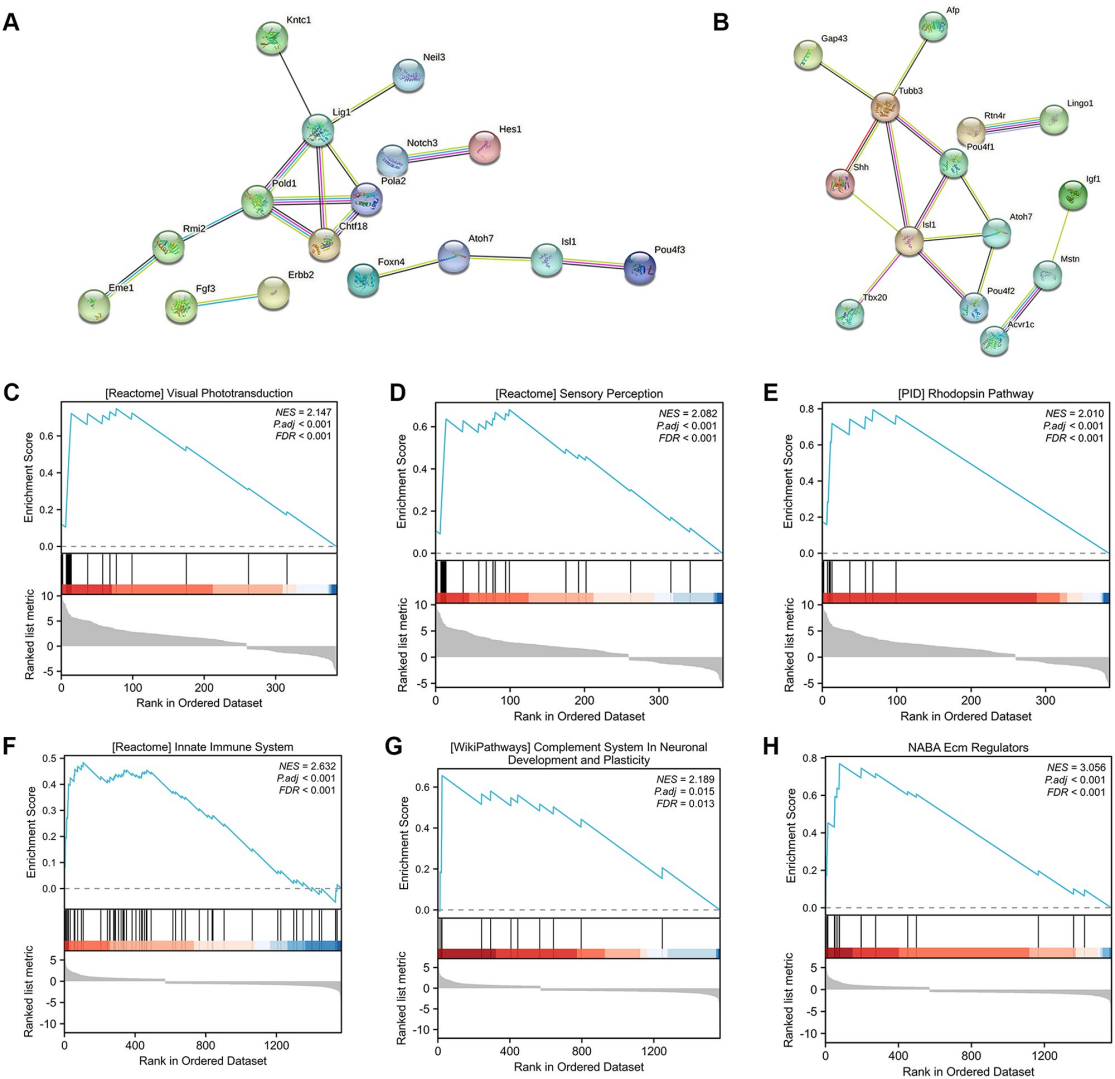
(A-B) Construction of PPI (protein-protein interaction) networks of age-DEGs and ONC-DEGs. Line color indicates the type of interaction evidence. (C-H) GSEA analyses of age-DEGs (C-E) and ONC-DEGs (F-H). The y-axis represents enrichment score, and the x-axis denotes genes (vertical black lines) represented in gene sets. The colored band at the bottom represents the degree of correlation of genes with the INR phenotype (red for positive and blue for negative correlation).

Gene expression data and protein-protein interaction (PPI) networks were used to identify the regulated parts of the network in DEGs. The network indicated both functional and physical protein associations. To identify the “communities” and “hubs” associated with the axon, we analyzed the cluster 7 in age-DEGs and cluster 5 in ONC-DEGs. The PPI networks for the two DEGs are shown in Fig 4A and 4B. The intersections of the age-DEGs resulted in the identification of the following hub genes, including *Pold1*, *Chtf18*, *Pola2* and *Lig1* (Fig 4A), and the hub genes of ONC-DEGs, including *Tubb3*, *Isl1* and *Pou4f1* (Fig 4B).

Moreover, GSEA analysis was performed on all age-DEGs and ONC-DEGs to find the involved pathways. GSEA detected the expression changes in sets of genes rather than individual genes, and showed additional pathways associated with the co-regulated genes. The age-DEGs presented as high expression included REACTOME_VISUAL_PHOTOTRANSDUCTION, REACTOME_SENSORY_PERCEPTION, and PID_RHODOPSIN_PATHWAY (Fig 4C–4E). REACTOME_INNATE_IMMUNE_SYSTEM, NABA_ECM_REGULATORS, and WP_COMPLEMENT_SYSTEM_IN_NEURONAL_DEVELOPMENT_AND_PLASTICITY were high expression in ONC-DEGs (Fig 4F–4H).

### RT-PCR validation of DEGs

**Fig 5:**
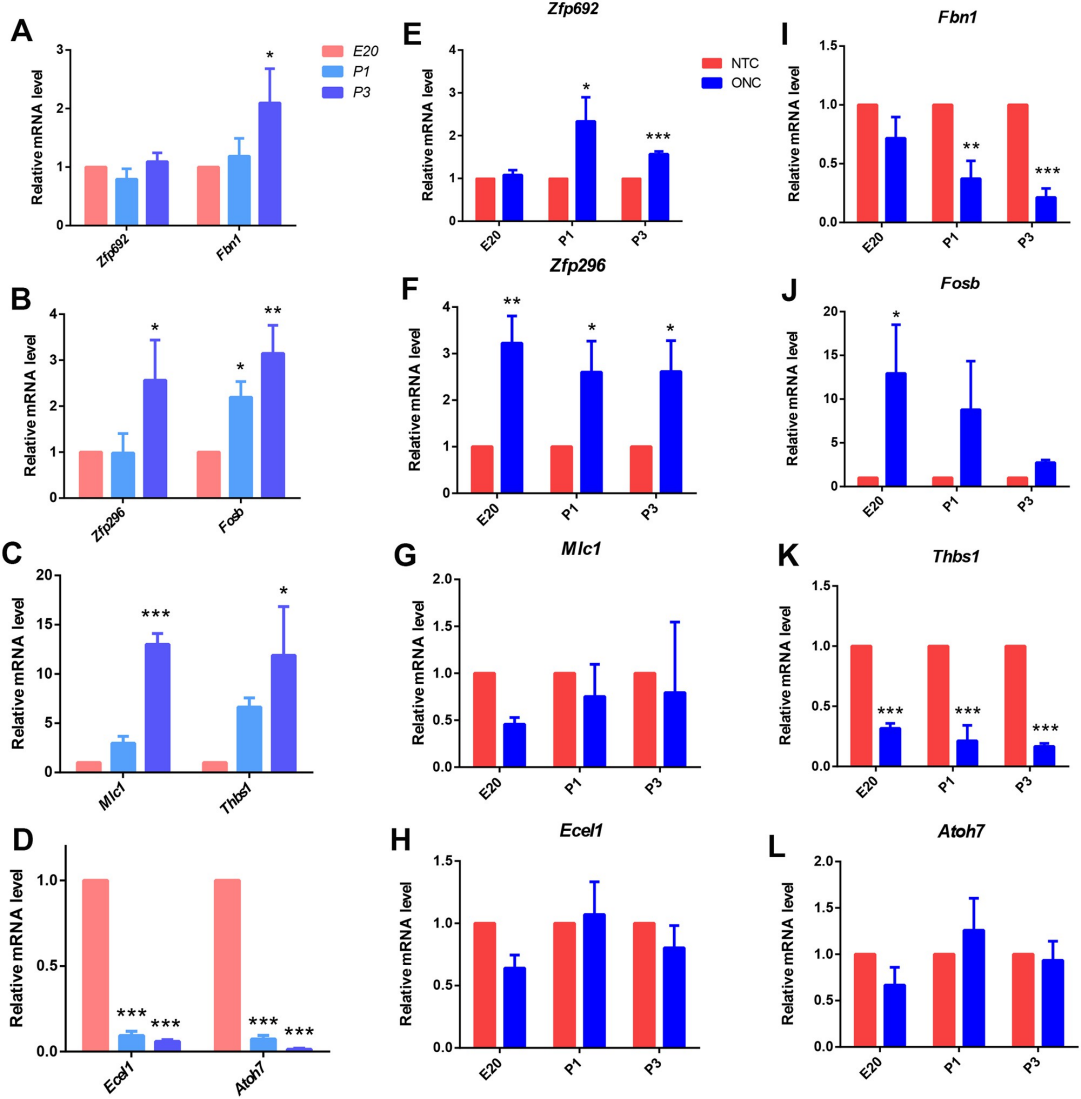
Validation of selected DEGs with qRT-PCR. **(A–D)** Statistical graph shows mRNA expression in the control groups with age, normalized to an average expression of 1.0 in the E20 group. **(E–L)** Statistical graph shows mRNA expression in the ONC and control groups, normalized to an average expression of 1.0 in the control group. Data are represented as Means ± SD; n = 3; *p < 0.05; **p < 0.01; ***p < 0.001.

To validate the RNA-Seq results, eight DEGs (*Mlc1*, *FosB*, *Zfp296*, *Thbs1*, *Fbn1*, *Fgf15、Ecel1* and *Atoh7*) were selected for expression validated by qRT-PCR. Significant differences in the expression of these eight genes were found between the NTC and ONC samples (Fig 5). This was consistent with the differential expression patterns observed in the RNA-Seq data.

## Discussion

In this study, we performed RNA-seq analysis on the normal and 6-hour post-ONC retinas from the E20, P1, and P3 mice to determine the retinal transcriptome profiles following axonal injury. The identified DEGs could be associated with the degeneration of axonal growth capacity after birth, and could be the novel genes (*Ecel1*, *Zfp296*, *Mlc1*, and *Fosb*) associated with axonal regeneration. Subsequent *K*‐means, GO, KEGG, PPI, and GSEA analysis revealed the biological processes and regulatory pathways involved in the DEGs.

During the initial formation of the visual projections, RGCs can extend the axons rapidly and regenerate the injured axons over a short distance [16]. The capacity for rapid axonal growth and regeneration decreases sharply within a day of birth [4], and the developmental decline is associated with numerous changes in gene expression [16]. Therefore, our study is based on the loss of axonal growth capacity after birth and analyzes the differences in the retinal transcriptome comparing with the normal and axonal damage before and after birth to identify the key genes associated with the axonal growth capacity.

We identified the DEGs that changed significantly after ONC, conducted a relevant literature search based on the differential multiplicity and gene description, and screened to identify 10 of these DEGs of interest in related to their age-related changes in the normal retina, including *Gstp*, *Mlc1*, *Pax2*, *Hnf4g*, *Cpne1*, *Egr1*, *Sall3*, *Sox1*, *Zfp692*, and *Zfp296*. *Mlc1* was one of the most significantly down-regulated genes in E20 ONC, but did not change in P1 and P3 ONC. In the normal retina, *Mlc1* expression increased significantly with age. *Mlc1* is mainly expressed in the ganglion cell layer. In the optic nerve, *Mlc1* is strongly expressed in a large number of astrocytes surrounding the axons [17]. No correlation between *Mlc1* and axonal regeneration has been reported. Combined with its changes in age and ONC groups, we suggest that the *Mlc1* gene could be a new target for axonal regeneration. *Zfp296* encodes a conserved mammalian factor belonging to the Cys2/ His2 type zinc finger (C2H2-ZF) family. The C2H2-ZF proteins have chromatin effects, affecting embryonic development by negatively regulating H3K9 methylation [18]. In addition, *Zfp296* binds to Oct3/4, Sox2, Klf4, and c-Myc expression to improve the efficiency of iPS cell generation [19]. Thus, the *Zfp296* gene could be a novel reprogramming factor for rescuing axonal injury.

Of the 415 DEGs that changed significantly with ages, *Atoh7* and *Ecel1* were the most down-regulated gene with ages. *Atoh7* is thought to be required to confer the ability to produce RGCs from retinal progenitor cells. Loss of function of *Atoh7*, expressed in multipotent early neurogenic retinal progenitors leads to a selective and essentially complete loss of RGCs [20]. *Ecel1* has been reported to promote antioxidant activity, and play a neuroprotective role in neurons [21]. The *Ecel1*-deficient mice failed to regenerate even after the zymosan treatment, which is a well-known regeneration-promoting reagent [22]. These suggest that *Ecel1* could be a new therapeutic strategy for axonal regeneration after ON injury.

For the analysis of differentially expressed transcription factors, *Creb5* was significantly upregulated in P3 NTC. This transcriptional regulator is active in response to noxious stressful stimuli and involved in cellular defense against stresses [23]. *Creb* has been reported to promote neuronal survival, and a recent study has shown that *Creb* is neuroprotective against hydrogen peroxide-induced RGC death through its two downstream cell survival genes [24], *Bdnf* and *Bcl-257*. Therefore, we suggest that *Creb5* is involved in axonal injury-induced RGC death, and is an important neuroprotective target in the treatment of axonal injury.

Interestingly, we found that the differential expression of some genes after ONC followed the same trend as differential expression with age, including *Fosb* and *Lcn2*. *Fosb* is a member of the *Fos* family of the AP-1 transcription factor complex, which encodes for the genes related to the neuronal activity [25]. *Fosb* was significantly upregulated after ONC and also significantly upregulated with ages in the normal retina. *Fosb* has been reported to be involved in morphological changes in neuronal synapses, and *Fosb* dysregulation will result in abnormal axonal branching [26]. In a spinal cord injury model, knockdown of *Fosb* increases the axon length in primary neurons cultured in *vitro* and activates the Stat3 pathway [27]; however, in another study, upregulation of *Fosb* expression enhances axon regeneration in mice after facial nerve axotomy [28], but there is no study related to ON regeneration yet. Therefore, the specific role of *Fosb* in ON axonal regeneration deserves further exploration. *Lcn2* is an acute stress response protein that involves in multiple pathways, such as inflammation, glial cell proliferation, cell differentiation, migration, and cell death [29]. *Lcn2* was significantly upregulated with ages and increased significantly in E20 ONC. Studies have shown that *Lcn2* expression is increased in the GCL layer after ONC [30], and in most cases the diversity of *Lcn2* functions may be attributed to its role as an iron-binding protein, and the *Lcn2*/iron-Bim pathway is thought to be closely associated with RGC apoptosis [31]. Thus, *Lcn2* may be involved in axonal injury-induced RGC death, and is a promising target for neuroprotection in the treatment of ON injury.

*K*-means analysis further divided DEGs into several clusters based on gene expression patterns. Clusters with similar expression patterns were enriched into the same terms in the GO analysis. The GO analysis identified that the age-related DEGs were mainly enriched in the visual perception, metabolic, DNA binding, axon guidance, and nerve development. The KEGG analysis revealed that the identified DEGs were enriched in phototransduction. These pathways were involved in the process of axon development, suggesting that these DEGs clusters could play the regulatory roles in age-related axonal growth. In the analysis of the ONC-related DEGs, the GO analysis suggested the DEGs were associated with the break repair, neuron projection guidance, and ganglion development. The KEGG analysis on the ONC-DEGs also enriched in axon guidance, implying cluster 1, 5, and 9 DEGs may be involved in axon regeneration after ONC. These suggest that these differential genes could be potential targets for optic nerve regeneration and repair.

In addition, we constructed the PPI networks for cluster 7 in age-DEGs and cluster 5 in ONC-DEGs, which were associated with axon. In the age-related cluster 7, gene expression decreased progressively with age. The hub genes *Pold1*, *Chtf18*, *Pola2* and *Lig1* are all DNA polymerases, and play a role in the early stages of DNA replication and DNA recombination [32, 33]. This may account for the rapid decline in axonal growth capacity early in life. The hub genes *Tubb3*, *Isl1* and *Pou4f1* in ONC-DEGs cluster 5 were expressed at reduced levels in E20-ONC. *Tubb3* belongs to the microtubule protein family, and plays a role in proper axon guidance and maintenance [34]. *Isl1* transcription factor plays an important role in the gene regulatory network of RGC differentiation [35], by cooperating with the transcription factor *Pou4f2* [36]. *Pou4f1* induces coordinated expression of neuronal protrusions and genes encoding synaptic proteins [37], and activates *Bcl2* to protect neuronal cells from apoptosis [38]. These hub genes could have significant implications for axonal regeneration after optic nerve injury, which may be potential targets for protection against RGC death after axonal injury.

According to the GSEA analysis, the age-related DEGs showed high expression in visual perception, in the same way in the enrichment analysis. The ONC-related DEGs presented high expression in the immune system pathway and neuronal development. The immune responses have been reported to be involved in CNS injury [39]. These results reveal the potential for axonal regeneration after ONC and the therapeutic targets.

In the present study, we performed the RNA-Seq and pathway analyses on the whole retinal samples, but not on the purified RGCs. The gene expression changes found in this study may represent other types of retinal cells in addition to RGCs. Furthermore, we did not predict the upstream regulators of key differential genes, and could not provide a more detailed understanding of the regulation of the DEGs. However, we combined multiple pathways and protein interaction network analysis to screen a number of candidate genes to provide new targets for a better understanding of the mechanisms underlying the changes in axonal growth capacity with ages and axonal regeneration after injury.

## Conclusions

This study, for the first time, delineated the gene expression changes in embryonic and neonatal mice following ON injury using RNA-Seq analysis to obtain a global view of retinal gene expression changes associated with the rapid changes in RGC growth capacity and axonal injury. A number of DEGs that may be involved in the rapid postnatal decline in RGC growth capacity and in axonal damage in the mouse retina were identified, with *Mlc1*, *Zfp296*, *Creb5*, *Ecel1*, *Fosb*, and *Lcn2* as the most promising candidates for resistance to axonal damage-induced RGC death and promotion of axonal regeneration and deserving further investigations. Although the analysis of the upstream transcriptional regulators of these DEGs still requires further investigations, this study provides new insights into the intrinsic molecular mechanisms for the axonal growth, offering a new resource for the age- and injury-driven studies on axonal growth capacity, and contributing to the discovery of axonal regeneration reprogramming factors after ON injury.

## Supporting information

S1 Table The sequence of the primers for qRT-PCR. (DOCX)

S2 Table Raw data. (XLSX)

S3 Table The RNA-seq QC data. (XLS)
