# Supplementary material for: Retinal transcriptome of neonatal mice after optic nerve injury
Source: PLoS One. 2023 May 30;18(5):e0286344. doi: 10.1371/journal.pone.0286344 (PMC10228772; doi:10.1371/journal.pone.0286344)
Supplement: S1 Table — (DOCX) [file pone.0286344.s001.docx]

**Supplementary table 1** The sequence of the primers for qRT-PCR.

| Gene | Forward primer (5‘-3’) | Reverse primer (5‘-3’) |
| --- | --- | --- |
| *Mlc1* | CCGAGATGGATTATTTGCGCTG | ATATCTGAAAGTTGGGAATCGCAC |
| *Zfp692* | CCAGGCTGTCCAGGAGAGTA | ATCTGGGGAGGAGCTGTAGG |
| *Fosb* | ATCGACTTCAGGCGGAAACTG | CTGGCAAATCTCTCACCTCGG |
| *Zfp296* | CCATCATCGCTTTCATGGATCAC | CAAGGACTAGTGTACTGTCTGCC |
| *Thbs1* | AGGACAGCATCCGAAAAGTGA | GATGGTAACCGAGTTCTGGCA |
| *Fbn1* | TGGAGAGGGCTGCATAGATGA | TCCGAAAAGCAGTACCCTTCC |
| *Ecel1* | TGGACAAATCCTCGTGGCTC | GTCAGTTCGTGCCCAATGATG |
| *Atoh7* | GGGTCTTAGGGGAGAAAACTCAATC | ACACAAGAGAAGTGGAGATAGGGA |
